# Corticosteroids as risk factor for COVID-19-associated pulmonary aspergillosis in intensive care patients

**DOI:** 10.1186/s13054-022-03902-8

**Published:** 2022-01-28

**Authors:** Rasmus Leistner, Lisa Schroeter, Thomas Adam, Denis Poddubnyy, Miriam Stegemann, Britta Siegmund, Friederike Maechler, Christine Geffers, Frank Schwab, Petra Gastmeier, Sascha Treskatsch, Stefan Angermair, Thomas Schneider

**Affiliations:** 1 Division of Gastroenterology, Infectious Diseases and Rheumatology, Medical Department, Charité–Universitätsmedizin Berlin, Corporate Member of Freie Universität Berlin, Humboldt-Universität zu Berlin and Berlin Institute of Health, Berlin, Germany; 2 Institute of Hygiene and Environmental Medicine, Charité–Universitätsmedizin Berlin, corporate member of Freie Universität Berlin, Humboldt-Universität zu Berlin and Berlin Institute of Health, Berlin, Germany; 3 Department of Anesthesiology and Intensive Care Medicine, Charité - Universitätsmedizin Berlin, Corporate Member of Freie Universität Berlin, Humboldt Universität zu Berlin, and Berlin Institute of Health, Berlin, Germany, Charité Campus Benjamin Franklin, Berlin, Germany; 4 Labor Berlin, Charité Vivantes GmbH, Berlin, Germany; 5 Department of Infectious Diseases and Respiratory Medicine, Charité - Universitätsmedizin Berlin, Corporate Member of Freie Universität Berlin, Humboldt-Universität zu Berlin, and Berlin Institute of Health, Berlin, Germany

**Keywords:** COVID-19, Pulmonary aspergillosis, CAPA, Dexamethasone, Corticosteroid

## Abstract

**Purpose:**

Corticosteroids, in particular dexamethasone, are one of the primary treatment options for critically ill COVID-19 patients. However, there are a growing number of cases that involve COVID-19-associated pulmonary aspergillosis (CAPA), and it is unclear whether dexamethasone represents a risk factor for CAPA. Our aim was to investigate a possible association of the recommended dexamethasone therapy with a risk of CAPA.

**Methods:**

We performed a study based on a cohort of COVID-19 patients treated in 2020 in our 13 intensive care units at Charité Universitätsmedizin Berlin. We used ECMM/ISHM criteria for the CAPA diagnosis and performed univariate and multivariable analyses of clinical parameters to identify risk factors that could result in a diagnosis of CAPA.

**Results:**

Altogether, among the *n* = 522 intensive care patients analyzed, *n* = 47 (9%) patients developed CAPA. CAPA patients had a higher simplified acute physiology score (SAPS) (64 vs. 53, *p* < 0.001) and higher levels of IL-6 (1,005 vs. 461, *p* < 0.008). They more often had severe acute respiratory distress syndrome (ARDS) (60% vs. 41%, *p* = 0.024), renal replacement therapy (60% vs. 41%, *p* = 0.024), and they were more likely to die (64% vs. 48%, *p* = 0.049). The multivariable analysis showed dexamethasone (OR 3.110, CI95 1.112–8.697) and SAPS (OR 1.063, CI95 1.028–1.098) to be independent risk factors for CAPA.

**Conclusion:**

In our study, dexamethasone therapy as recommended for COVID-19 was associated with a significant three times increase in the risk of CAPA.

***Trial registration*:**

Registration number DRKS00024578, Date of registration March 3rd, 2021.

**Supplementary Information:**

The online version contains supplementary material available at 10.1186/s13054-022-03902-8.

## Introduction

About < 5% of COVID-19 patients become critically ill [1]. International guidelines recommend corticosteroid therapy, for instance 6 mg dexamethasone systemically for 10 days, for patients in need of respiratory support [2]. As the length of the pandemic increases, a growing number of studies have reported COVID-19-associated pulmonary aspergillosis (CAPA) in these patients [3–9]. However, it is currently unclear whether the infection itself or therapeutic side-effects, e.g. those associated with corticosteroids, are responsible for the growing incidence of CAPA [10, 11].

Because critically ill COVID-19 patents frequently show signs of hyper inflammatory response, corticosteroid therapy has been shown to reduce overall mortality by 10–30% [12–15]. Corticosteroid therapy has, therefore, become an important pillar in COVID-19 treatment and is recommended by WHO, NIH, EMA and national guidelines [2, 16–18]. However, although corticosteroids suppress hyper inflammatory response, they also inhibit immune responses and pathogen clearance. Because COVID-19 is determined first by viral replication followed later by hyper inflammation, the timing of corticosteroid therapy is important [19, 20]. It has been shown that it is predominantly patients on respiratory support who benefit from corticosteroid therapy [14]; in contrast treatment performed too early might be associated with increased mortality [21, 22]. Moreover, corticosteroid therapy is a known risk factor for influenza-associated pulmonary aspergillosis (IAPA) [23].

Although the sheer number of reports of CAPA would illustrate the importance of this disease, there is currently a debate over diagnostic criteria and a potential overestimation of CAPA incidence [10, 24–26]. The current definition of invasive fungal disease, established by the European Organization for Research and Treatment of Cancer and the Mycoses Study Group Education and Research Consortium (EORTC/MSGERC), does not represent the clinical presentation of many CAPA patients [27]. Those criteria require a relevant, pre-existing immunodeficiency that are not present in most COVID-19 patients with suspected invasive pulmonary aspergillosis [24, 26]. Therefore, consensus criteria for CAPA, based on the altered requirements and clinically available diagnostic procedures, have been developed by the European Excellence Center for Medical Mycology and the International Society for Human and Animal Mycology (ECMM/ISHM) [25].

To date, the data on the effect of corticosteroid therapy as a risk factor for CAPA is insufficient and conflicting [11, 12, 14, 28–33]. Existing studies used various definitions of CAPA or did not perform multivariable analysis for this particular endpoint. Thus, we determined to examine the potential effect and effect size of corticosteroid therapy, applying ECMM/ISHM criteria as well as multivariable analysis that weighed competing risk factors for this endpoint.

## Methods

To analyze the risk of CAPA in intensive care, we collected data retrospectively from COVID-19 patients on intensive care units (ICU) at our university hospital, Charité – Universitätsmedizin Berlin, in 2020 (January, 1–December, 31). COVID-19 patients were defined as patients that had tested positive for SARS-CoV-2 by PCR and who had been admitted to an ICU specifically for treatment of COVID-19. We included all patients with COVID-19 who received intensive care in our facility. The cohort was based on a surveillance database provided by the Institute of Hygiene and Environmental Medicine. As part of its work on infection prevention, the institute conducts continuous, semi-automated pathogen surveillance, which in turn is based on routine microbiological and virological laboratory results [34]. Based on the assembled cohort, we searched the microbiology database for positive, culture-based detection of *Aspergillus spp.* and Aspergillus-specific antigen (galactomannan). A clinical microbiologist (TA) conducted this search and assisted in the validation process.

### CAPA-definition

The definition of CAPA was based on the 2020 ECMM/ISHAM consensus criteria for research and clinical guidance [25]. Broncho-alveolar lavage (BAL) and non-bronchoscopic lavage (NBL) were examined by direct microscopy or conventional mycological culture. Serum, broncho-alveolar lavage (BAL) and non-bronchoscopic lavage (NBL) were tested for Aspergillus-specific antigen (galactomannan) using the PLATELIA™ Aspergillus enzyme immunoassay (Bio-Rad, Marnes-la-Coquette, France) according to the manufacturer’s instructions. Positive results were fixed at a cut-off of > 0.5 for serum, ≥ 1.0 for BAL and ≥ 1.2 for NBL according to the consensus criteria [25]. PCR was not performed.

As suggested, ‘proven CAPA’ was defined as histopathological, direct microscopic or cultural detection of Aspergillus spp. directly from lung tissue, which is usually obtained post-mortem in our institution. The diagnosis of ‘possible CAPA’ or ‘probable CAPA’ required a combination of the same clinical, radiological criteria but differed in the additional microbiological criteria required. Clinical criteria included refractory fever, pleural rub, chest pain or haemoptysis. Refractory fever was defined as fever for more than 3 days or new fever after a period of defervescence for more than 48 h during adequate anti-infective therapy, without any other obvious cause. Radiologic criteria included a pulmonary infiltrate or nodules, preferably documented by chest CT, or cavitating infiltrate, not due to another cause. For ‘probable CAPA’, the microbiological requirements were BAL with Aspergillus spp. detection by direct microscopy, culture or positive antigen test or positive serum antigen test. For ‘possible CAPA’, the microbiological requirements were NBL with detection of Aspergillus spp. by direct microscopy, culture or positive antigen test.

### Specific analyzed parameter

To assess the risk factors for CAPA, the appropriate time-at-risk had to be determined. For CAPA cases, this was the period before first microbiological detection of *Aspergillus spp.* In the case of control patients, this was their entire time on ICU. Coinfections were assessed using CDC-based surveillance criteria [35].

We examined the administration of dexamethasone as categorical parameter as well as the number of days receiving systemic corticosteroid treatment after the admission to the ICU. In addition, we converted the different systemic corticosteroids administered to the corresponding cortisol dose in order to yield the cumulative dose during the time-at-risk. Finally, we evaluated whether a systemic corticosteroid medication was being used at the time of admission as a binary parameter.

Acute respiratory distress syndrome (ARDS) was classified using the Berlin definition [36]. The Charlson comorbidity index (CCI) was determined based on patients’ diagnosed comorbidities using the method of Charlson et al. [37] and on the adaptation to the ICD-10 of Thygesen et al. [38]. Severity of disease was assessed using the Simplified Acute Physiology Score (SAPS), as well as the laboratory parameters IL-6 and procalcitonin serum concentrations. These parameters were collected on admission (as baseline) as well as maximum value (as endpoint, representing the course of illness) during the patients’ stay on the ICU.

### Statistical analyses

Within our entire cohort, we identified CAPA cases and potential controls. We first analyzed the parameters that were available for the entire cohort and then performed another—more in-depth—nested case–control study. Therefore, we randomly selected 30% of the available controls for further data acquisition. In the randomization protocol, patients were first put in chronological order based on their date of admission to the hospital. Then every third patient was selected as a control. According to the literature, unlike most patients with invasive pulmonary aspergillosis, the current CAPA cases did not show signs of cancer or long term immunosuppression [25]. Nevertheless, to determine the risk factors leading to CAPA in these patients, we did not perform case–control matching but an exploratory case–control study.

Many cases were in the ICU for only a few days before being diagnosed with CAPA. A time-at-risk for CAPA that was too short would be associated with underreporting of relevant risk factors and, therefore, represented a systematic bias. Furthermore, there is no hospital-wide standard for the type and frequency of microbiological testing for *Aspergillus spp.* As a consequence, this could lead to under or over-reporting of CAPA cases, as discussed in literature [24]. To control for these biases, we performed sensitivity analyses on two further sub-cohorts. Univariate analyses were performed on the total case–control cohort (sub-cohort 1), as well as on patients with a minimum length of stay of five days (hereafter referred to as sub-cohort 2) and on patients with at least seven microbiological samples specific to *Aspergillus spp.* (hereafter referred to as sub-cohort 3).

In the descriptive univariate analyses, median and interquartile range (IQR) were calculated for the continuous parameters, number and percentage of binary parameters. Univariate differences were tested using the Wilcoxon rank-sum test for continuous variables and the Chi-square test for binary variables. All significance tests were two-tailed, and a *p* value of < 0.05 was considered significant. In addition, multivariable logistic regression was performed in order to assess the risk factors for CAPA. A *p* value of < 0.05 was considered significant. All analyses were conducted using SPSS (IBM SPSS statistics 27, Somer, NY, USA).

## Results

### Recruitment protocol

**Fig. 1:**
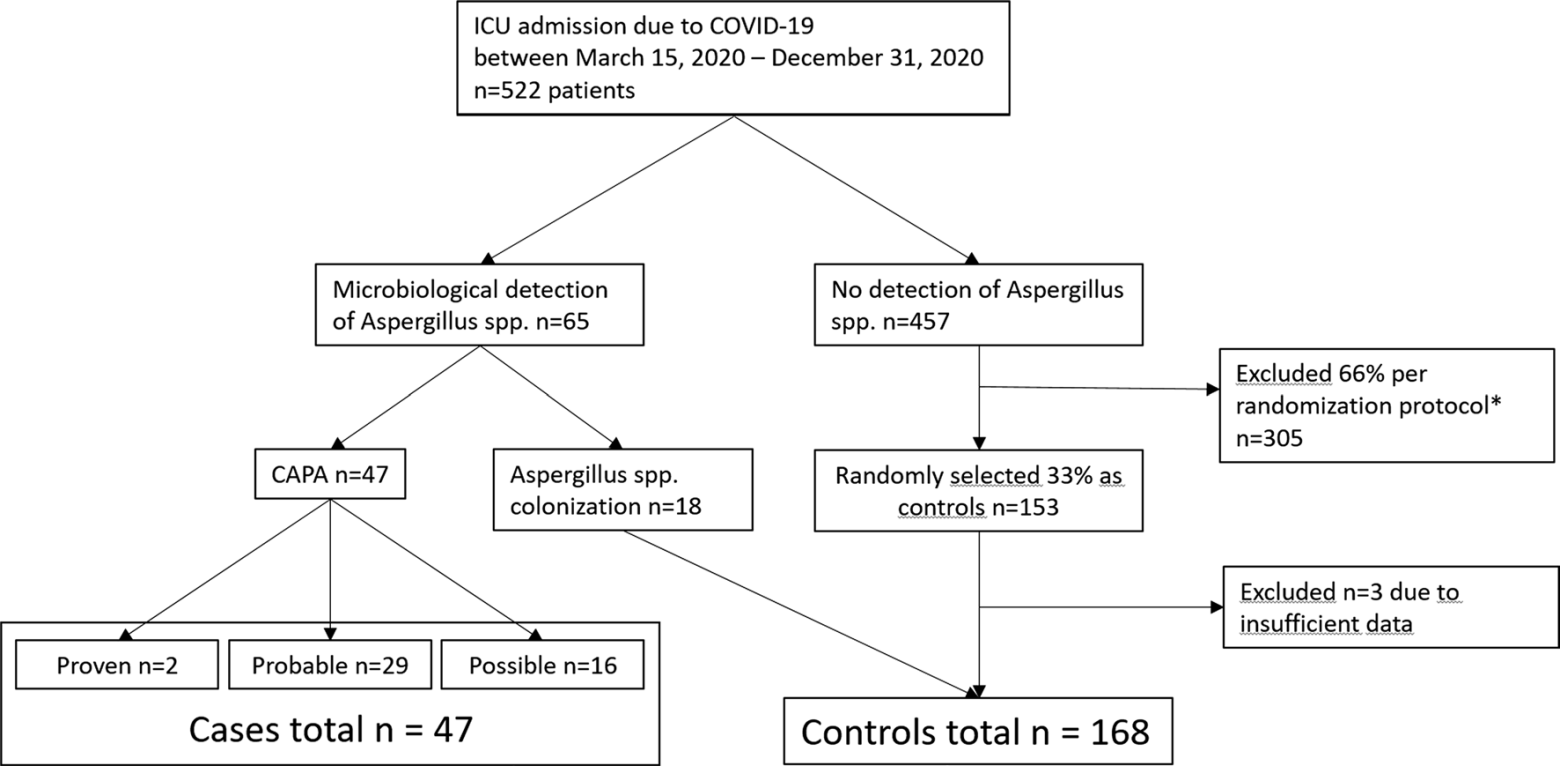
Recruitment flow chart for CAPA cases and controls. ICU, intensive care unit. CAPA, COVID-19 associated pulmonary aspergillosis. Proven, probable and possible CAPA based on the 2020 ECMM/ISHAM consensus criteria for research and clinical guidance

A total of 522 patients were admitted for COVID-19 to 13 different intensive care units in our institution during the study period. Over their course of illness, sixty-five (17.0%) patients were tested positive for *Aspergillus spp.* and underwent further examination to determine and verify the type of CAPA (proven, probable or possible) [25] (Fig. 1). Of the *n* = 457 patients without microbiological indication of *Aspergillus spp.,* *n* = 153 (33%) were selected as controls. Three control patients had to be excluded because of insufficient data. Within the cohort of potential CAPA case patients, *n* = 2 (3%) patients were classified as ‘proven,’ *n* = 29 (45%) as ‘probable’ and *n* = 16 (25%) as ‘possible CAPA’. Additional file 1: Table S1 shows that cases with possible CAPA had a more severe course of disease than did probable CAPA as reflected by higher SAPS scores and IL-6 serum concentrations. *N* = 18 (28%) patients who did not meet the CAPA criteria were classified as patients colonized by *Aspergillus spp*. and transferred to the control cohort (Table 1). In total, the case–control cohort consisted of *n* = 215 patients, *n* = 47 CAPA patients and *n* = 168 patients without CAPA (case–control-ratio 1:3.6). The univariate analysis did not show a relevant difference between included and excluded controls (Additional file 1: Table S3).

**Table 1:** Detailed classification of CAPA cases

	Aspergillus detected by mycological culture from BAL/NBL					Antigen detection		Radiological signs		Clinical signs			
	A. fumigatus	A. niger	A. flavus	A. nidulans	A. terreus	Ag Serum	Ag BAL/NBL	Pulmonary infiltrate	Cavitating infiltrate	Refractory fever	Pleural rub	Chest pain	Haemoptysis
Proven CAPA (*n* = 2)	100% (*n* = 2)	–	–	–	–	50% (*n* = 1)	50% (*n* = 1)	100% (*n* = 2)	–	50% (*n* = 1)	–	–	–
Probable CAPA (*n* = 29)	76% (*n* = 22)	10% (*n* = 3)	3% (*n* = 1)	3% (*n* = 1)	–	6% (*n* = 2)	66% (*n* = 19)	100% (*n* = 29)	6% (*n* = 2)	86% (*n* = 25)	–	3% (*n* = 1)	41% (*n* = 12)
Possible CAPA (*n* = 16)	81% (*n* = 13)	19% (*n* = 3)	–	–	–	–	–	100% (*n* = 16)	19% (*n* = 3)	69% (*n* = 11)	–	–	13% (*n* = 2)
Aspergillus spp. colonization (*n* = 18)	78% (*n* = 14)	–	–	–	6% (*n* = 1)	12% (*n* = 2)	24% (*n* = 4)	94% (*n* = 17)	6% (*n* = 1)	–	–	–	–

A., aspergillus. CAPA, COVID-19-associated pulmonary aspergillosis. BAL, bronchoalveolar lavage. NBL, non-bronchoscopic lavage. Ag, antigen

### Classification of CAPA cases

Detailed classification of CAPA cases, based on the definition of Koehler et al. [25], is shown in Table 1. Altogether, in the *n* = 47 verified CAPA cases, *A. fumigatus* (79%, *n* = 37) was the pathogen most frequently detected in cultures, followed by *A. niger* (13%) *n* = 6, *A. flavus* (2%, *n* = 1) and *A. nidulans* (2%, *n* = 1). Twenty cases (43%) exceeded the galactomannan cut-off in BAL or NBL, and three (6%) cases exceeded the cut-off in serum. The median onset (first microbiological detection as antigen or culture) was on day 8 following admission to the ICU (IQR 4–14).

CT scan of the chest found an infiltrate in all cases; a cavitating infiltrate was found in five (11%) cases. Seventy-nine % (*n* = 37) of cases had refractory fever, 40% (*n* = 19) showed hemoptysis, one (2%) patient had signs of chest pain, none were diagnosed with pleural rub.

The two ‘proven CAPA’ cases were diagnosed based on the autopsy report. Both had multiple white-greenish ulcers in the trachea and bronchi, suggesting super infection with fungi. Microscopic examination of lung tissue revealed fungal elements consistent with *Aspergillus spp.* and invasive growth into the lung tissue. In both cases, *A. fumigatus* was also detected in the mycological culture in the clinically obtained BAL or NBL specimen. Chest CT scans showed pulmonary infiltrates in both proven cases but no cavitating infiltrates. In one case, galactomannan was also detected in serum and BAL but without the required clinical signs. The galactomannan examination of the other case was negative for serum and BAL and showed refractory fever as the only clinical criterion.

Specific antifungal treatment (systemic triazoles, echinocandins or amphotericin B) for a minimum lengths of 14 days was administered in 23% of cases (Additional file 1: Table S2). Several CAPA patients showed further infections (coinfections). Sixty-six percent (*n* = 33) showed bacterial lower respiratory tract infection (LRTI), 17% (*n* = 8) had a blood stream infection, and 9% (*n* = 4) had a urinary tract infection. In 70% of LRTI cases (*n* = 23), the pathogen found by BAL was: *Klebsiella spp.* *n* = 5, *Staphylococcus aureus* *n* = 5, *Streptococcus pneumoniae* *n* = 3, *E. coli* *n* = 3, *Enterobacter spp.* *n* = 3, *Pseudomonas aeruginosa* *n* = 2, *Acinetobacter baumanii* *n* = 2.

### Timeline for the 2020 pandemic

**Fig. 2:**
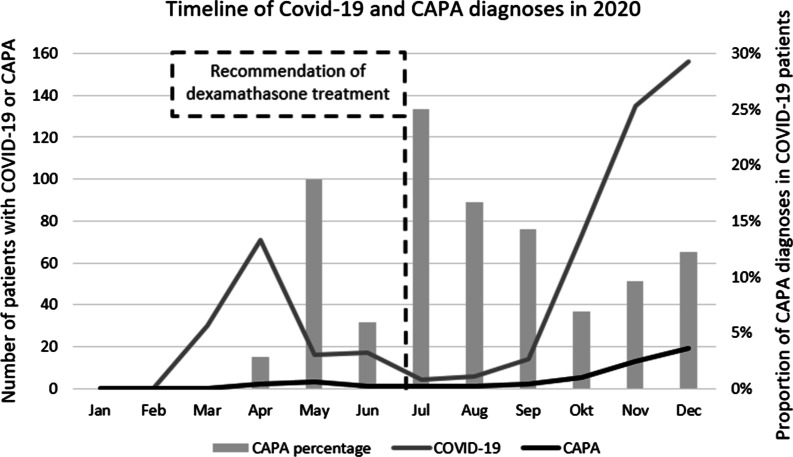
Time course of COVID-19 and CAPA diagnoses in 2020. The line chart shows the number of COVID-19 admissions to our ICUs (grey) and the CAPA cases below (black). The bar chart in the background shows the percentage of CAPA cases within monthly admissions. The doted vertical line indicates the point at which the hospital-wide recommendation for early dexamethasone treatment (6 mg, once daily for 10 days) was introduced

In 2020, increasing numbers of patients with COVID-19 and respiratory insufficiency were admitted to our intensive care units with the first cases appearing in March. As the pandemic progressed, the number of patients with COVID-19 declined in the summer but rallied again in autumn and peaked in December during the so-called second wave (Fig. 2). The number of CAPA cases also increased in late 2020 during this second wave. In July 2020, dexamethasone treatment (6 mg, once daily for 10 days) was introduced as the hospital-wide standard for the early treatment of critically ill COVID-19 patients, based on international recommendations [2, 15]. We found an overall CAPA incidence of 9% (47/522) in our total cohort of COVID-19 ICU patients. Up to July, we diagnosed 4.5% patients with CAPA (*n* = 6/*n* = 134) and from July to December 10.6% (*n* = 41/*n* = 388). This sharp increase in CAPA cases was statistically significant (*p* = 0.049 chi square, RR 4.286, CI95% 1.779–9.234). Tocilizumab was not recommended at the time, and we found only 3 patients who had been or were being treated with it in the control cohort.

Table 2 shows univariate comparisons of relevant endpoints for CAPA cases and controls. Sixty-four % of the CAPA cases died during their hospital stay, compared to 48% of the control patients. Parameters representing the severity of illness during disease course (LOS on ICU, SAPS maximum, IL-6 maximum, rates of severe ARDS) were in CAPA cases significantly higher than in controls. The results were stable in the sub-cohorts (Additional file 1: Table S4).

**Table 2:** Univariate comparison of endpoint parameters in CAPA cases and controls

Parameter		Total Case Control Cohort (*n* = 215)		
		Control (*n* = 168)	Cases (*n* = 47)	*p *value
LOS Hospital (days)		24 (IQR 12–48)	33 (IQR 19–53)	**0.033**
LOS Intensive Care (days)		20 (IQR 7–42)	24 (IQR 17–43)	**0.020**
SAPS maximum		53 (IQR 40–65)	64 (IQR 50–69)	**0.001**
IL-6 maximum (ng/l)		461.4 (IQR 133–1634)	1005 (IQR 203–4789)	**0.008**
PCT maximum (µg/l)		3.23 (IQR 0.64–13.7)	7.48 (IQR 3.95–16.27)	**0.012**
ECMO		20.8% (*n* = 35)	21.3% (*n* = 10)	0.947
ARDS	None	18.45% (*n* = 31)	*n* = 0	**0.007**
	Mild	1.8% (*n* = 3)	2.1% (*n* = 1)	
	Moderate	19.6% (*n* = 33)	14.9% (*n* = 7)	
	Severe	60.1% (*n* = 101)	83.0% (*n* = 39)	
In-hospital death		47.6% (*n* = 80)	63.8% (*n* = 30)	**0.049**
Renal replacement therapy		41.1% (*n* = 69)	59.6% (*n* = 28)	**0.024**

*P*-value ≤ 0.05 was defined as statistically significant and presented as bold

LOS, length of stay; PCT, procalcitonin; SAPS, simplified acute physiology score; IL-6, interleukin-6; ECMO, extra-corporeal membrane oxygenation; ARDS, acute respiratory distress syndrome; IQR, inter quartile range

Table 3 shows univariate comparisons of potential risk factors for CAPA in cases and controls. CAPA cases were significantly more likely to receive invasive ventilation during risk period, had higher SAPS scores on admission, suffered from chronic kidney disease more often, had more microbiological samples of *Aspergillus spp.*, and higher Charlson comorbidity scores. However, CAPA cases admitted received pre-existing corticosteroid therapy less frequently and had lower daily corticosteroid doses during their stay in ICU. Though not statistically significant, the percentage of CAPA cases with dexamethasone therapy was 11% above the rate of controls. The univariate analysis of sub-cohorts 2 and 3 showed similar, thus stable, results (Additional file 1: Table S5). All CAPA patients were mechanically ventilated whereas only 75% (*n* = 126) of control patients. The univariate comparison of only mechanically ventilated patients showed higher in-hospital mortality in CAPA patients, though not statistically significant (63.8% vs. 54.8%, *p* = 0.284) while the other parameter showed similar results as the full analysis (Additional file 1: Table S6).

**Table 3:** Univariate comparison of potential risk factors for CAPA in cases and controls

Parameters	Total Cohort investigated (*n* = 215)		
	Control (*n* = 168)	Cases (*n* = 47)	*p* value
*Parameters on admission*			
Age (years)	65.5 (IQR 55.5–75.1)	67.4 (IQR 62.4–75.9)	0.105
Male gender	75% (*n* = 126)	87% (*n* = 41)	0.075
BMI (kg/m^2^)	29 (IQR 25.5–32)	30 (IQR 26–34)	0.499
SAPS	41 (IQR 32–48)	51 (IQR 44–59)	** < 0.001**
Lymphcytes/nl	0.83 (IQR 0.56–1.22)	0.77 (IQR 0.38–1.06)	0.112
Neutrophils/nl	7.94 (IQR 5.51–11.13)	8.14 (IQR 5.55–11.94)	0.781
IL-6 (ng/l)	110 (IQR 34.3–315.4)	91.65 (IQR 58.3–215)	0.868
CRP (mg/l)	142.3 (IQR 68.9–234.3)	160.8 (IQR 108.6–270.9)	0.167
PCT (µg/l)	0.38 (IQR 0.15–1.49)	0.71 (IQR 0.22–2)	0.169
Corticosteroids on admission	8% (*n* = 10)	21% (*n* = 24)	** < 0.001**
Admission from external ICU	42% (*n* = 71)	21% (*n* = 24)	0.283
*ICU treatment parameters*			
Number of microbiological samples for *Aspergillus spp.*	3 (IQR 0–7)	7 (IQR 5–11)	**0.000**
Length of ICU stay before onset of CAPA (days)	Not applicable	8 (IQR 4–14)	Not applicable
Dexamethasone therapy	76.2% (*n* = 128)	87.2% (*n* = 41)	0.103
Cortisol cumulative dose (mg)	1.470 (390–2.670)	900 (200–2.200)	0.154
Corticosteroid treatment (days)	7 (IQR 2–12)	6 (IQR 2–10)	0.298
Invasive ventilation (days)	12 (IQR 1–32)	23 (IQR 16–38)	** < 0.001**
Days without mechanical ventilation	1 (IQR 0–6)	1 (IQR 0–5)	0.171
*Comorbidities*			
Charlson Comorbidity Index	5 (IQR 3–7)	6 (IQR 5–8)	**0.015**
Peptic ulcer	2% (*n* = 4)	4% (*n* = 2)	0.490
Rheumatoid disease	4% (*n* = 7)	4% (*n* = 2)	0.979
Heart disease	24% (*n* = 41)	15% (*n* = 7)	0.166
Vascular disease	15% (*n* = 25)	17% (*n* = 8)	0.719
Diabetes	31% (52)	32% (*n* = 15)	0.900
Liver disease	20% (*n* = 33)	23% (*n* = 11)	0.572
Renal disease	65% (*n* = 109)	87% (*n* = 41)	**0.003**
Cancer	5% (*n* = 9)	11% (*n* = 5)	0.195
AIDS/HIV	0 (*n* = 0)	0 (*n* = 0)	n.s
Neurological disease	8% (*n* = 14)	6% (*n* = 3)	0.661
Lung disease	29% (*n* = 49)	40% (*n* = 19)	0.142

*P*-value ≤ 0.05 was defined as statistically significant and presented as bold

CAPA, COVID-19-associated pulmonary aspergillosis; LOS, length of stay; BMI, body mass index; ICU, intensive care unit; PCT, procalcitonin; SOFA, sequential organ failure assessment; SAPS, simplified acute physiology score; IL-6, interleukine-6; CRP, C-reactive protein; IQR, inter quartile range

### Multivariable logistic regression

Based on the results of univariate analysis and risk factors for CAPA cited in the literature [9, 11, 22, 33], the following parameters were included in the multivariable analysis: Dexamethasone therapy, corticosteroids on admission, age, male gender, SAPS on admission, BMI, lung disease, diabetes, renal disease and cancer.

Table 4 shows the results of two multivariable analyses for risk factors for CAPA in the entire case–control cohort and in the sub-cohort of patients that were mechanically ventilated (*n* = 126 controls, *n* = 47 CAPA cases). Dexamethasone therapy, SAPS and days with invasive ventilation were independently and directly associated with an increased risk for CAPA. *R*^2^ for the models, stated as Nagelgerkes *R*^2^, were 0.272 and 0.219. We furthermore performed a multivariable analysis for in-hospital mortality (Table 5). Severe ARDS and Charlson comorbidity index were independently associated with in-hospital mortality.

**Table 4:** Results of the multivariable analyses for risk factors for CAPA

	Cases control cohort * n* = 215				Mechanically ventilated patients only * n* = 173			
	*p* value	OR	95% CI for OR		*p* value	OR	95% CI for OR	
			Lower	Upper			Lower	Upper
Male gender	0.068	2.564	0.932	7.051	0.160	2.102	0.745	5.929
Age (years)	0.490	1.012	0.978	1.047	0.208	1.024	0.987	1.062
Dexamethasone therapy	**0.031**	**3.110**	**1.112**	**8.697**	**0.036**	**3.039**	**1.076**	**8.583**
BMI (kg/m^2^)	0.409	1.024	0.968	1.083	0.320	1.031	0.971	1.094
SAPS on admission	**0.000**	**1.063**	**1.028**	**1.098**	**0.002**	**1.055**	**1.020**	**1.091**
Corticosteroids on admission	0.099	2.373	0.850	6.622	0.211	1.927	0.690	5.384
Lung disease	0.437	1.352	0.632	2.894	0.483	1.319	0.608	2.859
Renal disease	0.059	2.722	0.963	7.693	0.170	2.136	0.722	6.323
Diabetes	0.981	0.991	0.446	2.199	0.890	0.944	0.418	2.132
Cancer	0.375	1.794	0.493	6.524	0.426	1.697	0.462	6.240

*P*-value ≤ 0.05 was defined as statistically significant and presented as bold

SAPS, simplified acute physiology score; OR, odds ratio; BMI, body mass index

**Table 5:** Results of the multivariable analysis for risk factors for in-hospital mortality

	*p* value	OR	95% CI for OR	
			Lower	Upper
Male	0.621	1.215	0.562	2.626
Age	0.848	1.003	0.974	1.032
BMI	0.742	0.992	0.946	1.040
SAPS	0.150	1.021	0.992	1.051
CAPA	0.945	1.028	0.466	2.270
Charlson Comorbidity Index	**0.004**	**1.245**	**1.071**	**1.448**
ARDS milde	0.999	0.000	0.000	0.000
ARDS moderate	0.858	1.120	0.324	3.867
ARDS severe	**0.025**	**3.640**	**1.174**	**11.289**

*P*-value ≤ 0.05 was defined as statistically significant and presented as bold

CAPA, COVID-19 associated pulmonary aspergillosis; SAPS, simplified acute physiology score; BMI, body mass index; ARDS, acute respiratory distress syndrome

## Discussion

The internationally recommended dexamethasone therapy is an important element in the treatment of patients with severe or critical COVID-19 [14, 15, 39]. However, as more cases of COVID-19-associated pulmonary aspergillosis (CAPA) are reported, the treatment is suspected to pose a relevant risk of fungal super infection, such as CAPA [11, 40].

### Corticosteroid therapy

We found that dexamethasone therapy as recommended mediated a significantly increased risk of CAPA. Several other studies showed that corticosteroid therapy can be associated with increased risk of CAPA [29–32, 41, 42]. However, those studies identified very different cortisol dosages, treatment lengths and indications. Two systematic meta-analyses found long-term corticosteroid treatment upon admission to be a relevant risk factor for CAPA [4, 9]. In our univariate analysis, we found an association between corticosteroid treatment before admission and CAPA. However, this risk factor was no independent factor in the multivariable model. Nevertheless, it is known from the treatment of influenza patients that the administration of corticosteroids on admission or during intensive care is an increased risk for invasive pulmonary aspergillosis [20].

Even though, corticosteroids have a strong beneficial effect on the 28 day mortality [12], systemic corticosteroid therapy can hamper pathogen clearance and potentially pose a relevant risk during viral replication, e.g. in early COVID-19 [11, 21, 24]. In a recently published French multicenter study, dexamethasone and anti-IL-6 together were associated with a threefold increased risk of CAPA [40]. However, they could not estimate the effect of dexamethasone alone. In the meta-analysis on the effect of corticosteroids on survival in critically ill COVID-19 patients, Sterne et al. argued that the optimal dose and duration of treatment could not be sufficiently assessed. They explicitly stated that it is unclear whether a lower dose of corticosteroids would be associated with lower benefit [15]. Our results therefore underline that the current dose of corticosteroid therapy in critically ill COVID-19 patients should be reviewed in further studies.

### Burden of CAPA

The overall incidence of CAPA in our cohort was 9% (47/522; 6% proven/probable CAPA, 3% possible CAPA).

In an attempt to harmonize the various incidences available, Fekkar et al. applied the ECMM/ISHAM criteria retrospectively to currently available studies [10]. They found an overall incidence of 10% (128/1288; 6.9% proven/probable CAPA and 3.1% possible CAPA). Thus, our results strongly support the currently available estimation on CAPA incidence.

### Other risk factors

Other published risk factors for CAPA were hematological malignancy [30], solid organ transplantation [31], low body mass index [4, 30], chronic kidney disease or renal replacement therapy [4, 43] and chronic lung disease [22, 30, 32, 43] or disease severity [31]. No relevant influence of gender, age or diabetes was found [4, 29]. Our results support renal diseases and disease severity as risk factors for CAPA. After all they are indicators for an overall deterioration in physical condition.

We found that invasive ventilation was a relevant risk factor for CAPA which is known as important risk factors for ventilator-associated pneumonia (VAP) in general [44]. Nevertheless, one study compared ventilated COVID-19 patients with non-COVID-19 ventilated patients and found an increased incidence overall of VAP (including CAPA) associated with COVID-19 [45]. Another author then suggested early prophylaxis with antifungal agents for critically ill COVID-19 patients [22]. Our data support the findings that the onset of CAPA is about one week after admission to the ICU [22, 24]. However, within our 13 analyzed ICUs we could not find an indication for an environmental factor. Eventually, appropriate strategies have to be found to decrease the risk of CAPA. Treating critical ill COVID-19 patients with antifungal agents upon ICU admission regardless other risk factors might not be an appropriate strategy.

### Mortality

Out data show higher in-hospital mortality associated with CAPA than in non-CAPA controls (64% vs. 48%), although this was not verified in our multivariable analyses, the results support the findings of earlier studies [4, 8]. Whereas, a CAPA-associated mortality was found between 46 and 55% [8, 22, 43], the overall estimated mortality among critically ill COVID-19 patients is approximately 40% [15, 46]. Even though our numbers are higher compared to previously published figures, they are within the given confidence intervals of their results. As a tertiary care center with specialized ECMO ICUs in a metropolitan area, we may have treated a selection of highly critical. This is demonstrated by the fact that 44% of our patients were transferred from external ICUs, and the SAPS on admission were on average as high as 41. Hence, the estimated mortality rates from our study appear plausible and support the current body of evidence on increased mortality from CAPA.

### Limitations

Our study has limitations. It is a retrospectively conducted assessment. Hence, potential underlying biases such as availability of data, availability of diagnostic procedures and treatments performed during clinical routine could have an influence on the results observed. However, we conducted multiple sub-cohort analyses in order to control for this effect. We found no relevant bias; our results were stable. Because resources were limited, we were only able to analyze parts of the control cohort as a nested-case–control study. However, the controls were randomly selected. The comparison of the basic parameters between included and excluded controls revealed no relevant differences. This underscores once more the validity of our results.

## Conclusion

In this study of severe and critical ill COVID-19 patients, dexamethasone therapy was associated with an increased risk of pulmonary aspergillosis and mortality. Nonetheless, corticosteroid therapy in these patients improves the overall mortality after 28 days. However, our results show that about 10.6% of ICU patients with severe or critical COVID-19, and the currently recommended dexamethasone regimen develop CAPA compared to 4.5% without this therapy. We need further studies to evaluate the appropriate corticosteroid dose and length to weigh the benefits and harms of this therapy.

## Supplementary Information

**Additional file 1**.** Supplementary material**.** Supplementary Table S1**. Univariate comparison of proven, probable and possible CAPA.** Supplementary Table S2**. Antifungal treatment and coinfections in CAPA cases.** Supplementary Table S3**. Univariate comparison between included and excluded controls.** Supplementary Table S4**. Univariate comparison of endpoint parameters in CAPA cases and controls for total case control cohort, sub cohort 2 and sub cohort 3.** Supplementary Table S5**. Univariate comparison of potential risk factors for CAPA in cases and controls for total case control cohort, sub cohort 2 and sub cohort 3.** Supplementary Table S6**. univariate comparison of cases, controls and mechanically ventilated controls.

## Data Availability

The datasets used and/or analysed during the current study are available from the corresponding author on reasonable request.
