# Supplementary material for: Corticosteroids as risk factor for COVID-19-associated pulmonary aspergillosis in intensive care patients
Source: Crit Care. 2022 Jan 28;26:30. doi: 10.1186/s13054-022-03902-8 (PMC8796178; doi:10.1186/s13054-022-03902-8)
Supplement: Supplementary file 1 — Additional file 1. Supplementary material. Supplementary Table S1. Univariate comparison of proven, probable and possible CAPA. Supplementary Table S2. Antifungal treatment and coinfections in CAPA cases. Supplementary Table S3. Univariate comparison between included and excluded controls. Supplementary Table S4. Univariate comparison of endpoint parameters in CAPA cases and controls for total case control cohort, sub cohort 2 and sub cohort 3. Supplementary Table S5. Univariate comparison of potential risk factors for CAPA in cases and controls for total case control cohort, sub cohort 2 and sub cohort 3. Supplementary Table S6. univariate comparison of cases, controls and mechanically ventilated controls. [file 13054_2022_3902_MOESM1_ESM.docx]

Supplementary material:

Supplementary Table S1: Univariate comparison of proven, probable and possible CAPA.

Supplementary Table S2: Antifungal treatment and coinfections in CAPA cases.

Supplementary Table S3: Univariate comparison between included and excluded controls.

Supplementary table S4: Univariate comparison of endpoint parameters in CAPA cases and controls for total case control cohort, sub cohort 2 and sub cohort 3.

Supplementary Table S5: Univariate comparison of potential risk factors for CAPA in cases and controls for total case control cohort, sub cohort 2 and sub cohort 3.

Supplementary Table S6: univariate comparison of cases, controls and mechanically ventilated controls

**Supplementary table S1: Univariate comparison of proven, probable and possible CAPA**

|  | | **Proven (n=2)** | **Probable (n=29)** | **Possible (n=16)** | **P-value** |
| --- | --- | --- | --- | --- | --- |
| **ARDS** | **ARDS mild** | 0 (n=0) | 3% (n=1) | 0 (n=0) | 0.584 |
|  | **ARDS moderate** | 0 (n=0) | 21% (n=6) | 6% (n=1) |  |
|  | **ARDS severe** | 1 (n=2) | 76% (n=22) | 94% (n=15) |  |
| **Male gender** | | 1 (n=2) | 83% (n=24) | 94% (n=15) | 0.491 |
| **Admission from external ICU** | | 50% (n=1) | 48% (n=14) | 56% (n=9) | 0.877 |
| **Death** | | 100% (n=2) | 62% (n=18) | 56% (n=9) | 0.486 |
| **Corticosteroid on admission** | | 0 (n=0) | 21% (n=6) | 13% (n=2) | 0.632 |
| **Invasive ventilation (days)** | | 25 (IQR 17-33) | 23 (IQR 16-34) | 30 (IQR 14-42) | 0.834 |
| **Dexamethasone therapy** | | 100% (n=2) | 90% (26) | 81% (13) | 0.619 |
| **Cortisol cumulative dose (mg)** | | 360 (IQR 0-1080) | 825 (IQR 0-1.650) | 1.050 (IQR 450-1.970) | 0.732 |
| **Corticosteroid treatment (days)** | | 3 (IQR 0-7) | 6 (0-11) | 7 (4-10) | 0.439 |
| **Renal replacement therapy** | | 50% (n=1) | 55% (n=16) | 69% (n=11) | 0.648 |
| **ECMO** | | 0 (n=0) | 17% (n=5) | 31% (n=5) | 0.412 |
| **Age (years)** | | 70.7 (IQR 67.9-73.5) | 66.4 (IQR 63.1-73.8) | 66.5 (IQR 58.3-78) | 0.879 |
| **LOS hospital (days)** | | 26 (IQR 17-34) | 31 (IQR 20-56) | 41 (IQR 19-53) | 0.712 |
| **LOS ICU (days)** | | 25 (IQR 17-33) | 24 (IQR 17-36) | 29 (IQR 15-51) | 0.842 |
| **Length of ICU stay before onset of CAPA (days)** | | 7 (IQR 0-14) | 8 (IQR 5-14) | 8 (IQR 2-27) | 0.956 |
| **Number of microbiological samples** | | 4 (IQR 2-6) | 8 (IQR 5-11) | 7 (IQR 4-11) | 0.828 |
| **BMI (kg/m²)** | | 25.3 (IQR 24.6-25.9) | 29.9 (IQR 26.1-32.6) | 32.2 (IQR 25.3-35.5) | 0.515 |
| **SAPS on admission** | | 52 (IQR 47-56) | 49 (IQR 42-55) | 58 (IQR 49-66) | **0.030** |
| **Lymphocytes/nl on admission** | | 0.4 (IQR 0.4-0.5) | 0.7 (IQR 0.4-1) | 0.8 (IQR 0.2-1) | 0.828 |
| **SAPS maximum** | | 64 (IQR 61-67) | 63 (IQR 56-68) | 70 (IQR 64-74) | **0.006** |
| **IL-6 maximum (ng/l)** | | 28,249 (IQR 6,499-50,000) | 742 (IQR 175-4,600) | 2,586 (IQR 373-12,255) | 0.225 |
| **PCT maximum (µg/l)** | | 30.4 (IQR 12.6-48.3) | 5.2 (IQR 1.5-11) | 8.9 (IQR 6.9-17.2) | **0.035** |
| **Neutrophils/nl on admission** | | 13.7 (IQR 11-16.5) | 8.1 (IQR 6.3-12.4) | 7.4 (IQR 3.4-9.8) | 0.243 |
| **IL-6 (ng/l) on admission** | | 416.5 (IQR 150-683) | 70.9 (IQR 47.3-117.3) | 147.5 (IQR 87.2-353.3) | **0.003** |
| **CRP (mg/l) on admission** | | 104 (IQR 60.5-147.5) | 160 (IQR 116-219.3) | 177.3 (IQR 111.7-298.5) | 0.474 |
| **PCT (µg/l) on admission** | | 1.6 (IQR 0.4-2.7) | 0.6 (IQR 0.1-1.2) | 1.6 (IQR 0.2-2.8) | 0.067 |
| **Charlson comorbidity index** | | 9 (IQR 7-10; range 0-0) | 6 (IQR 5-7; range 0-0) | 6 (IQR 5-9; range 0-0) | 0.523 |

**Supplementary table S2: Antifungal treatment and coinfections in CAPA cases.**

|  |  | **CAPA cases (n=47)** |
| --- | --- | --- |
| Treatment | **Antifungal resistance tested** | 70% (n=33) |
|  | **Appropriate antifungal treatment*** | 23% (n=11) |
|  | **Amphotericin B** | 45% (n=5) |
|  | **Triazole** | 36% (n=4) |
|  | **Echinocandin** | 82% (n=9) |
| Coinfections | **Cumulative number of coinfections** | n=76 |
|  | **Median number of coinfections** | 1 (IQR 1-3) |
|  | **Blood stream infection** | 17% (n=8) |
|  | **Lower respiratory tract infection** | 66% (n=31) |
|  | **Urinary tract infection** | 9% (n=4) |

Appropriate antifungal treatment, systemic antifungal agent (triazole, echinocandin or amphotericin B) for at least 14days. IQR, interquartile range.

**Supplementary table S3: Univariate comparison between included and excluded controls**

|  | Included Controls | Excluded Controls | P-Value |
| --- | --- | --- | --- |
| Male gender | 75% (n=126) | 66% (n=202) | 0.038 |
| Age (years) | 66 (IQR 56-75) | 65 (IQR 55-77) | 0.931 |
| Charlson comorbidity index | 5 (IQR 3-7) | 5 (IQR 3-7) | 0.225 |
| In-hospital death | 48% (n=80) | 39% (n=119) | 0.061 |

IQR, interquartile range.

**Supplementary table S4:** Univariate comparison of endpoint parameters in CAPA cases and controls.

| **Parameter** | | **Total Case Control Cohort (n=215)** | | | **Sub cohort with an examined time at risk > 4 days (n=182)** | | | **Sub cohort 3: Patients with > 6 microbiological samples (n=73)** | | |
| --- | --- | --- | --- | --- | --- | --- | --- | --- | --- | --- |
|  |  | **Control (n=168)** | **Cases (n=47)** | **P-value** | **Control (n=146)** | **Cases (n=36)** | **P-value** | **Control (n=48)** | **Cases (n=25)** | **P-value** |
| **LOS Hospital (days)** | | 24 (IQR 12-47.5) | 33 (IQR 19-53) | **0.033** | 29 (IQR 14-51) | 34 (IQR 23-57) | 0.064 | 55 (IQR 42-79) | 53 (IQR 34-81) | 0.356 |
| **LOS Intensive Care (days)** | | 20 (IQR 7-41.5) | 24 (IQR 17-43) | **0.020** | 23 (IQR 11-44) | 29 (IQR 18.5-48.5) | 0.052 | 53.5 (IQR 39.5-66) | 42 (IQR 25-58) | 0.087 |
| **SAPS maximum** | | 53 (IQR 40-65) | 64 (IQR 50-69) | **0.001** | 54 (IQR 43-66) | **65 (IQR 57-70)** | **0.004** | 64 (IQR 52-68) | 66 (IQR 63-69) | **0.081** |
| **IL-6 maximum (ng/l)** | | 461.4 (IQR 133-1,634) | 1,005 (IQR 203-4,789) | **0.008** | 553.5 (IQR 199-2,214) | 1820 (IQR 386.25-6,479.5) | **0.002** | 688 (IQR 405.3-3,863) | 3,418 (IQR 732.1-4,956) | 0.091 |
| **PCT maximum(µg/l)** | | 3.23 (IQR 0.64-13.7) | 7.48 (IQR 3.95-16.27) | **0.012** | 4.17 (IQR 0.83-14) | 8.37 (IQR 4.07-17.23) | **0.038** | 8.07 (IQR 2.38-20.6) | 9.06 (IQR 4.17-20.95) | 0.636 |
| **ECMO** | | 21% (n=35) | 21 % (n=10) | 0.947 | 23% (n=33) | 22% (n=8) | 0.961 | 42% (n=20) | 24% (n=6) | 0.247 |
| **ARDS** | **none** | 18 % (n=31) | n=0 | **0.007** | 10.27% (n=15) | n=0 | **0.023** | n=0 | n=0 | 0.396 |
|  | **mild** | 2% (n=3) | 2% (n=1) |  | 1% (n=2) | n=0 |  | n=0 | n=0 |  |
|  | **moderate** | 20% (n=33) | 15% (n=7) |  | 22% (n=32) | 8 % (n=3) |  | 6% (n=3) | 12% (n=3) |  |
|  | **severe** | 60 % (n=101) | 83% (n=39) |  | 66% (n=97) | 92% (n=33) |  | 94% (n=45) | 88% (n=22) |  |
| **In-hospital death** | | 48% (n=80) | 64% (n=30) | **0.049** | 51% (n=745) | 64% (n=23) | 0.155 | 48% (n=23) | 64% (n=16) | 0.104 |
| **Renal replacement therapy** | | 41% (n=69) | 60% (n=28) | **0.024** | 45% (n=65) | 67% (n=24) | **0.017** | 58% (n=28) | 76% (n=19) | 0.137 |

LOS, length of stay. PCT, procalcitonin. SAPS, simplified acute physiology score. IL-6, interleukin-6. ECMO, extra-corporeal membrane oxygenation. ARDS, acute respiratory distress syndrome. IQR, inter quartile range.

**Supplementary table S5:** Comparison of potential risk factors for CAPA in CAPA cases and controls without CAPA

| **Parameter** | **Total Case Control Cohort (n=215)** | | | **Sub cohort 2: Patients with time at risk > 4 days (n=182)** | | | **Sub cohort 3: Patients with > 6 microbiological samples (n=73)** | | |
| --- | --- | --- | --- | --- | --- | --- | --- | --- | --- |
|  | **Control (n=168)** | **Cases (n=47)** | **P-value** | **Control (n=146)** | **Cases (n=36)** | **P-value** | **Control (n=48)** | **Cases (n=25)** | **P-value** |
| **Number of microbiological samples for Aspergillus spp.** | 3 (IQR 0-7) | 7 (IQR 5-11) | **0.000** | 4 (IQR 1-8) | 8.5 (IQR 5.5-11.5) | **0.000** | 11 (IQR 8-14.5) | 10 (IQR 9-14) | 0.774 |
| **Age (years)** | 66 (IQR 55-75) | 67 (IQR 62-75) | 0.105 | 64.73 (IQR 55.48-75) | 66.94 (IQR 62.86-75.67) | 0.099 | 62.65 (IQR 57.54-71.46) | 66.45 (IQR 63.18-75.35) | 0.081 |
| **BMI (kg(m²)** | 29 (IQR 26-32) | 30 (IQR 26-34) | 0.499 | 29 (IQR 25-33) | 30.5 (IQR 26-33.5) | 0.542 | 28 (IQR 25-33.5) | 29 (IQR 26-33) | 0.761 |
| **SAPS on admission** | 41 (IQR 32-48) | 51 (IQR 44-59) | **0.000** | 41 (IQR 33-48) | 51 (IQR 46-59) | **0.000** | 42 (IQR 35-46) | 52 (IQR 48-62) | **0.000** |
| **Lymphocytes/nl on admission** | 0.83 (IQR 0.56-1.22) | 0.77 (IQR 0.38-1.06) | 0.112 | 0.82 (IQR 0.54-1.19) | 0.77 (IQR 0.34-1.05) | 0.260 | 0.82 (IQR 0.51-1.25) | 0.82 (IQR 0.48-1.05) | 0.376 |
| **Neutrophils/nl on admission** | 7.9 (IQR 5.5-11.1) | 8.1 (IQR 5.6-11.9) | 0.781 | 8.39 (IQR 5.91-11.39) | 8.23 (IQR 5.9-11.74) | 0.711 | 9.43 (IQR 7.61-11.36) | 8.11 (IQR 6.36-10.26) | 0.178 |
| **IL-6 (ng/l) on admission** | 110 (IQR 34-315) | 91.7 (IQR 58-215) | 0.868 | 116 (IQR 45.35-320.9) | 85.9 (IQR 56.8-251) | 0.694 | 198.7 (IQR 63.8-389.3) | 85.9 (IQR 56.8-251) | 0.220 |
| **CRP (mg/l) on admission** | 142.3 (IQR 68.9-234.3) | 160.8 (IQR 108.6-270.9) | 0.167 | 145.75 (IQR 82.1-242.15) | 161.15 (IQR 117.25-270.65) | 0.377 | 181.6 (IQR 118.5-264.9) | 161.5 (IQR 93-270.4) | 0.557 |
| **PCT(µg/l) on admission** | 0.38 (IQR 0.15-1.49) | 0.71 (IQR 0.22-2.00) | 0.169 | 0.39 (IQR 0.19-1.49) | 0.86 (IQR 0.19-2.09) | 0.318 | 0.38 (IQR 0.21-1.67) | 0.71 (IQR 0.15-2) | 0.575 |
| **Dexamethasone therapy** | 76% (n=128) | 87% (n=41) | 0.103 | 75.3% (n=110) | 86.1% (n=31) | 0.166 | 64.4% (n=31) | 80.0% (20) | 0.173 |
| **Cortisol cumulative dose (mg)** | 1.470 (390-2.670) | 900 (200-2.200) | 0.154 | 1.800 (IQR 900-2940) | 1.500 (IQR 870-2478) | **0.492** | 3.177 (IQR 1.175-5.980) | 1.500 (900-2.575) | **0.009** |
| **Average cortisol dose per cortisol treatment day** | 180 (IQR 140-207) | 150 (IQR 50-215) | **0.039** | 180 (IQR 156-215) | 150 (IQR 143.3-219.65) | 0.183 | 180 (IQR 132.41-235.76) | 150 (IQR 136.6-218.88) | 0.370 |
| **Days of invasive ventilation** | 12 (IQR 1-32) | 23 (IQR 16-38) | **<0.001** | 18 (6-35) | 29 (18-49) | 0.002 | 45 (IQR 28-60) | 36 (IQR 24-58) | 0.531 |
| **Charlson Comorbidity Index** | 5 (IQR 3-7) | 6 (IQR 5-8) | **0.015** | 5.5 (IQR 3-8) | 6 (IQR 5-7.5) | 0.052 | 6 (IQR 4-8. range 0-18) | 6 (IQR 5-8. range 0-10) | 0.430 |
| **Corticosteroid on admission** | 8% (n=10) | 21% (n=24) | **<0.001** | 10% (n=14) | 22% (n=8) | **0.037** | 15% (n=7) | 12% (n=3) | 0.761 |
| **Myocardial infarction** | 24% (n=4) | 21% (n=1) | 0.919 | 1% (n=1) | 3% (n=1) | 0.281 | 0 (n=0) | 4% (n=1) | 0.105 |
| **Congestive heart failure** | 23% (n=38) | 15% (n=7) | 0.250 | 25% (n=36) | 19% (n=7) | 0.510 | 23% (n=11) | 24% (n=6) | 0.994 |
| **Peripheral vascular disease** | 8% (n=13) | 2% (n=1) | 0.168 | 8% (n=11) | 3% (n=1) | 0.303 | 8% (n=4) | 4% (n=1) | 0.577 |
| **Male** | 75% (n=126) | 87% (n=41) | 0.075 | 77% (n=112) | 89% (n=32) | 0.107 | 90% (n=43) | 96% (n=24) | **0.013** |
| **Admission from external ICU** | 42% (n=71) | 51% (n=24) | 0.283 | 45% (n=66) | 47% (n=17) | 0.828 | 52% (n=25) | 60% (n=15) | 0.519 |
| **Peptic ulcer** | 2% (n=4) | 4% (n=2) | 0.490 | 3% (n=4) | 6% (n=2) | 0.397 | 8% (n=4) | 4% (n=1) | 0.778 |
| **Rheumatoid disease** | 4% (n=7) | 4% (n=2) | 0.979 | 5% (n=7) | 6% (n=2) | 0.850 | 4% (n=2) | 4% (n=1) | 0.676 |
| **Mild liver disease** | 7% (n=11) | 6% (n=3) | 0.968 | 7% (n=10) | 6% (n=2) | 0.779 | 4% (n=2) | 8% (n=2) | 0.762 |
| **Moderate to severe liver disease** | 17% (n=28) | 17% (n=8) | 0.954 | 20% (n=28) | 17% (n=6) | 0.729 | 17% (n=8) | 16% (n=4) | 0.989 |
| **Diabetes without complications** | 28% (n=47) | 26% (n=12) | 0.740 | 28% (n=41) | 19% (n=7) | 0.292 | 25% (n=12) | 24% (n=6) | 0.354 |
| **Diabetes with complications** | 3% (n=5) | 6% (n=3) | 0.275 | 3% (n=5) | 8% (n=3) | 0.198 | 4% (n=2) | 8% (n=2) | 0.788 |
| **Renal disease** | 65% (n=109) | 87% (n=41) | **0.003** | 69% (n=101) | 92% (n=33) | **0.006** | 73% (n=35) | 84% (n=21) | 0.477 |
| **Malign tumor** | 2% (n=4) | 6% (n=3) | 0.172 | 3% (n=4) | 6% (n=2) | 0.397 | 2% (n=1) | 4% (n=1) | 0.333 |
| **Metastatic solid tumour** | 2% (n=4) | 2% (n=1) | 0.919 | 3% (n=4) | 0 (n=0) | 0.315 | 4% (n=2) | 0 (n=0) | 0.152 |
| **AIDS/HIV** | 0 (n=0) | 0 (n=0) | n.s. | 0 (n=0) | 0 (n=0) | n.s. | 0 (n=0) | 0 (n=0) | n.s. |
| **Leukaemia** | 1% (n=2) | 2% (n=1) | 0.628 | 1% (n=2) | 3% (n=1) | 0.552 | 04% (n=2) | 0 (n=0) | 0.460 |
| **Lymphoma** | 1% (n=2) | 2% (n=1) | 0.628 | 1% (n=2) | 3% (n=1) | 0.552 | 4% (n=2) | 0 (n=0) | 0.152 |
| **Dementia** | 4% (n=7) | 2% (n=1) | 0.514 | 4% (n=6) | 0 (n=0) | 0.216 | 2% (n=1) | 0 (n=0) | 0.393 |
| **Hemiplegia** | 4% (n=7) | 4% (n=2) | 0.979 | 4% (n=6) | 6% (n=2) | 0.705 | 8% (n=4) | 8% (n=2) | 0.446 |
| **Lung disease** | 29% (n=49) | 40% (n=19) | 0.142 | 32% (n=47) | 44% (n=16) | 0.166 | 42% (n=20) | 52% (n=13) | 0.536 |

**Supplementary Table S6: univariate comparison of cases, controls and mechanically ventilated controls**

| **Parameters** | **Total Cohort investigated (n=215)** | | |  | **Only mechanically ventilated** | |
| --- | --- | --- | --- | --- | --- | --- |
|  | **Control (n=168)** | **Cases (n=47)** | **P-value** |  | **Controls (n=126)** | **P-value** |
| **Parameters on admission** | | | | | | |
| Age (years) | 65.5 (IQR 55.5-75.1) | 67.4 (IQR 62.4-75.9) | 0.105 |  | 63,9 (IQR =54,7-73,2) | **0,029** |
| Male gender | 75% (n=126) | 87% (n=41) | 0.075 |  | 79% (n=100) | 0,236 |
| BMI (kg/m²) | 29 (IQR 25.5-32) | 30 (IQR 26-34) | 0.499 |  | 29 (IQR =25-33) | 0,651 |
| SAPS | 41 (IQR 32-48) | 51 (IQR 44-59) | **<0.001** |  | 42 (IQR =35-51) | **<0.001** |
| Lymphcytes/nl | 0.83 (IQR 0.56-1.22) | 0.77 (IQR 0.38-1.06) | 0.112 |  | 0,8 (IQR =0,5-1,3) | 0,120 |
| Neutrophils/nl | 7.94 (IQR 5.51-11.13) | 8.14 (IQR 5.55-11.94) | 0.781 |  | 9,1 (IQR =6,3-11,7) | 0,278 |
| IL-6 (ng/l) | 110 (IQR 34.3-315.4) | 91.65 (IQR 58.3-215) | 0.868 |  | 151,5 (IQR =65,8-394,2) | 0,147 |
| CRP (mg/l) | 142.3 (IQR 68.9-234.3) | 160.8 (IQR 108.6-270.9) | 0.167 |  | 156 (IQR =97,2-259,2) | 0,999 |
| PCT (µg/l) | 0.38 (IQR 0.15-1.49) | 0.71 (IQR 0.22-2) | 0.169 |  | 0,5 (IQR =0,2-2,2) | 0,717 |
| Corticosteroids on admission | 8% (n=10) | 21% (n=24) | **<0.001** |  | 10% (n=13) | 0,059 |
| Admission from external ICU | 42% (n=71) | 21% (n=24) | 0.283 |  | 55% (n=69) | 0,664 |
| **ICU treatment parameters** | | | | | | |
| Number of microbiological samples for *Aspergillus spp.* | 3 (IQR 0-7) | 7 (IQR 5-11) | **0.000** |  | 5 (IQR =2-9) | **0,011** |
| Length of ICU stay before onset of CAPA (days) | Not applicable | 8 (IQR 4-14) | Not applicable |  | Not applicable | Not applicable |
| Dexamethasone therapy | 76.2% (n=128) | 87.2% (n=41) | 0.103 |  | 74.6% (n=94) | 0,074 |
| Cortisol cumulative dose (mg) | 1.470 (390-2.670) | 900 (200-2.200) | 0.154 |  | 1800 (IQR =1000-3286) | **0,003** |
| Corticosteroid treatment (days) | 7 (IQR 2-12) | 6 (IQR 2-10) | 0.298 |  | 10 (IQR =4-16) | **0,011** |
| Invasive ventilation (days) | 12 (IQR 1-32) | 23 (IQR 16-38) | **<0.001** |  | 20 (IQR =9-41) | 0,239 |
| **Comorbidities** | | | | | | |
| Charlson Comorbidity Index | 5 (IQR 3-7) | 6 (IQR 5-8) | **0.015** |  | 6 (IQR =3-8) | 0,096 |
| Peptic ulcer | 2% (n=4) | 4% (n=2) | 0.490 |  | 3% (n=4) | 0,730 |
| Rheumatoid disease | 4% (n=7) | 4% (n=2) | 0.979 |  | 4% (n=5) | 0,932 |
| Heart disease | 24% (n=41) | 15% (n=7) | 0.166 |  | 25% (n=31) | 0,170 |
| Vascular disease | 15% (n=25) | 17% (n=8) | 0.719 |  | 14% (n=18) | 0,654 |
| Diabetes | 31% (52) | 32% (n=15) | 0.900 |  | 31% (n=39) | 0,903 |
| Liver disease | 20% (n=33) | 23% (n=11) | 0.572 |  | 25% (n=31) | 0,870 |
| Renal disease | 65% (n=109) | 87% (n=41) | **0.003** |  | 77% (n=97) | 0,135 |
| Cancer | 5% (n=9) | 11% (n=5) | 0.195 |  | 6% (n=8) | 0,341 |
| AIDS/HIV | 0 (n=0) | 0 (n=0) | n.s. |  | 0 (n=0) | n.s. |
| Neurological disease | 8% (n=14) | 6% (n=3) | 0.661 |  | 7% (n=9) | 0,861 |
| Lung disease | 29% (n=49) | 40% (n=19) | 0.142 |  | 34% (n=43) | 0,442 |
